# Novel Solid Self-Nanoemulsifying Drug Delivery System (S-SNEDDS) for Oral Delivery of Olmesartan Medoxomil: Design, Formulation, Pharmacokinetic and Bioavailability Evaluation

**DOI:** 10.3390/pharmaceutics8030020

**Published:** 2016-06-27

**Authors:** Ali Nasr, Ahmed Gardouh, Mamdouh Ghorab

**Affiliations:** 1 Department of Pharmaceutics, Faculty of Pharmacy and Pharmaceutical Industry, Sinai University, Alarish 45511, Egypt; 2 Department of Pharmaceutics, Faculty of Pharmacy, Suez Canal University, Ismailia 41111, Egypt; Ahmed_mahmoud@pharm.suez.edu.eg (A.G.); mghorab@hotmail.com (M.G.)

**Keywords:** Olmesartan, solid self-nanoemulsifying drug delivery system (S-SNEDDS), Capryol 90, Cremophor RH40, Transcutol HP, Aerosil 200, spray drying

## Abstract

The main purpose of this study was to develop a solid self-nanoemulsifying drug delivery system (S-SNEDDS) of Olmesartan (OLM) for enhancement of its solubility and dissolution rate. In this study, liquid SNEDDS containing Olmesartan was formulated and further developed into a solid form by the spray drying technique using Aerosil 200 as a solid carrier. Based on the preliminary screening of different unloaded SNEDDS formulae, eight formulae of OLM loaded SNEEDS were prepared using Capryol 90, Cremophor RH40 and Transcutol HP as oil, surfactant and cosurfactant, respectively. Results showed that the mean droplet size of all reconstituted SNEDDS was found to be in the nanometric range (14.91–22.97 nm) with optimum PDI values (0.036–0.241). All formulae also showed rapid emulsification time (15.46 ± 1.34–24.17 ± 1.47 s), good optical clarity (98.33% ± 0.16%–99.87% ± 0.31%) and high drug loading efficiency (96.41% ± 1.20%–99.65% ± 1.11%). TEM analysis revealed the formation of spherical and homogeneous droplets with a size smaller than 50 nm. In vitro release of OLM from SNEDDS formulae showed that more than 90% of OLM released in approximately 90 min. Optimized SNEDDS formulae were selected to be developed into S-SNEDDS using the spray drying technique. The prepared S-SNEDDS formulae were evaluated for flow properties, differential scanning calorimetry (DSC), scanning electron microscopy (SEM), reconstitution properties, drug content and in vitro dissolution study. It was found that S-SNEDDS formulae showed good flow properties and high drug content. Reconstitution properties of S-SNEDDS showed spontaneous self-nanoemulsification and no sign of phase separation. DSC thermograms revealed that OLM was in solubilized form and FTIR supported these findings. SEM photographs showed smooth uniform surface of S-SNEDDS with less aggregation. Results of the in vitro drug release showed that there was great enhancement in the dissolution rate of OLM. To clarify the possible improvement in pharmacokinetic behavior of OLM S-SNEDDS, plasma concentration-time curve profiles of OLM after the oral administration of optimized S-SNEDDS formula (F3) were compared to marketed product and pure drug in suspension. At all time points, it was observed that OLM plasma concentrations in rats treated with S-SNEDDS were significantly higher than those treated with the drug in suspension and marketed product.

## 1. Introduction

The oral delivery route is the most convenient and preferred route for drug administration to achieve desired therapeutic effects and the greatest degree of patient compliance, especially for chronic condition diseases [1]. However, most new drug candidates and many existing drug molecules show poor aqueous solubility which leads to poor oral bioavailability, high intra- and inter-subject variability and lack of dose proportionality [2]. Despite some clinical oral formulations having been developed, the low oral bioavailability of most drugs is still a major hurdle leading to challenges for pharmaceutical manufacturers to design delivery systems that can provide improved pharmacokinetic profiles and therapeutic responses [3].

An ideal oral drug delivery system must protect the drug from the degradation in the gastrointestinal tract and deliver the bioactive compounds to the specific area where it is better absorbed. Because of this, considerable effort has been made in the improvement of oral drug delivery, drug stability in the GIT, increasing drug solubility and furthering the bioavailability [4]. A range of novel strategies are currently being developed for efficient delivery of poor water-soluble drugs. Among all these, the most accepted strategy is the lipid-based formulation approach [5].

In fact, the most popular strategy is the incorporation of the drug molecule into inert lipid vehicles such as oils and surfactant dispersions, self-emulsifying formulations, emulsions and liposomes, with particular emphasis on self-nanoemulsifying drug delivery systems (SNEDDS) [6]. Self-nanoemulsifying drug delivery systems (SNEDDS) are isotropic mixtures of oil, surfactant, cosurfactant and the drug that form fine oil-in-water (o/w) nanoemulsion when introduced into aqueous phases under gentle agitation. SNEDDS spread readily in the gastrointestinal tract and the digestive motility of the stomach and the intestine provide the agitation necessary for self-emulsification [7].

Upon administration, the isotropic mixture will come in contact with the aqueous phase of gastrointestinal tract and form an oil-in-water nanoemulsion with the aid of gastrointestinal motility. This spontaneous formation of nanoemulsion in the gastrointestinal tract presents the drug in solubilized form inside small droplets of oil, all over its transit through the GIT [8]. The nano-sized droplets also provide a large interfacial surface area for drug release and absorption. Conventional SNEDDS are usually prepared as liquid dosage forms that can be administered in soft gelatin capsules [9], which have some limitations such as: high production cost, incompatibility problems with capsule shell [10], low drug portability and stability, drug leakage and precipitation, low drug loading, few choices of dosage forms and irreversible drugs/excipients precipitation. More importantly, the large quantity of surfactants in the formulations can induce gastrointestinal irritations [11].

In recent years, much attention has been paid to solid self-nanoemulsifying drug delivery systems (S-SNEDDS), which have shown reasonable successes in improving oral bioavailability of poorly soluble drugs [12]. This drug delivery system combines the advantages of liquid SNEDDS with those of a solid dosage form and overcomes the limitations associated with liquid formulations [13]. S-SNEDDS also exhibited more commercial potential and patient acceptability [14]. Many techniques are offered to convert conventional liquid SNEDDS to solid form such as spray drying, adsorptions to solid carriers, spray cooling, melt extrusion, melt granulation, supercritical fluid based methods and high pressure homogenization. The resulting powder may then be filled directly into hard gelatin capsules or mixed with suitable excipients before compression into tablets.

**Figure 1:**
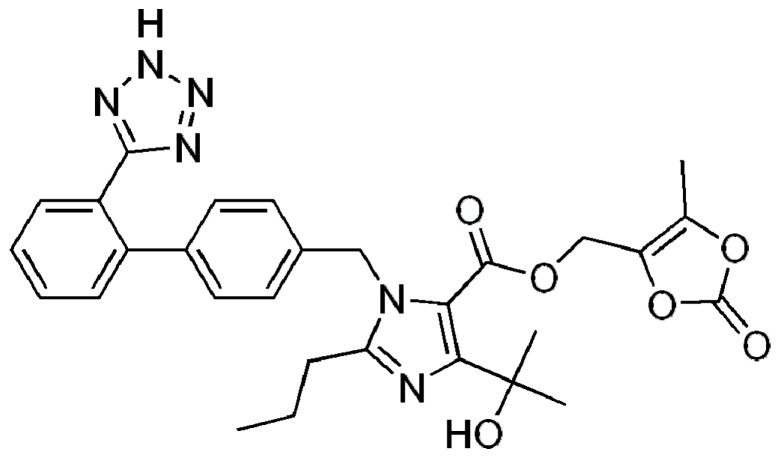
Chemical Structure of Olmesartan Medoxomil.

Olmesartan Medoxomil is a novel selective angiotensin II receptor blocker that is approved for the treatment of hypertension. OLM is a poorly water soluble drug and its aqueous solubility is reported to be less than 1 mg/mL due to its hydrophobic nature, as presented in Figure 1. It is a prodrug that is rapidly de-esterified during absorption from the gastrointestinal tract to produce an active metabolite. The oral bioavailability of OLM is only 26% in healthy humans due to its low solubility in water and unfavorable breakage of the ester drug to a poorly permeable parent molecule in the gastrointestinal fluids [15]. To overcome the problems concerning OLM, there was a need to develop S-SNEDDS which improves the oral bioavailability of OLM. Hence, the present study aimed towards the development of S-SNEDDS of OLM by a spray drying technique using Aerosil 200 as solid carrier for enhanced oral bioavailability.

## 2. Experimental Section

### 2.1. Materials

Olmesartan (Jedco International Pharmaceuticals, Cairo, Egypt), Miglyol 812, Miglyol 818, Miglyol 829 and Labrafil M 1944 CS (gift from Medical Union Pharmaceuticals, Ismailia, Egypt), Capryol 90, Gelucire 44/14, Lauroglycol FCC, Labrafac lipophile WL 1349, Maisine 35-1 and Transcutol HP (Gattefossé, Saint-Priest, Lyon, France), Cremophor RH40, Cremophor S9 and Labrasol (Nerol Chemicals, Cairo, Egypt), Bitter almond oil, Castor oil, Olive oil, Cotton seed oil, Arachis oil, Oleic acid, Hydrochloric acid and Propylene Glycol (El-Nasr Pharmaceutical Chemicals, Cairo, Egypt), Tween 20, Tween 40, Tween 60, Tween 80, Span 20, Span 80, PEG 400, PEG 600 and Sodium Hydroxide (Oxford Laboratory, Maharashtra, India), PEG 200 (Loba Chem. Pvt. Ltd., Mumbai, India), Glycerin (El-Gomhouria Pharmaceuticals, Cairo, Egypt) and Sodium Dihydrogen Phosphate (PureLab, Madison, WI, USA). Other chemicals are of HPLC grade.

### 2.2. Methods

#### 2.2.1. Preformulation Study (Selection of SNEDDS Components)

##### Study of OLM Solubility in Various Oils, Surfactants and Cosurfactants

In order to find out the right SNEDDS components with good solubilizing capacity for OLM, saturation solubility was performed on different oils (Gelucire 44/14, Lauroglycol FCC, Labrafac lipophile WL 1349, Capryol 90, Labrafil M 1944 CS, Miglyol 812, Miglyol 818, Miglyol 829, Maisine 35-1, Bitter almond oil, Castor oil, Olive oil, Cotton seed oil, Arachis oil and Oleic acid), surfactants (Cremophor RH40, Cremophor S9, Labrasol, Tween 20, Tween 40, Tween 60, Tween 80, Span 20 and Span 80) and cosurfactants (Transcutol HP, PEG 200, PEG 400, PEG 600, Propylene glycol and Glycerin) using the shake flask method [16]. In this study, an excess amount of the drug (approximately 500 mg) was introduced into 2 mL of each vehicle in screw capped greiner tubes. The mixtures were mixed well using a vortex mixer (MaxiMix II, Orlando, FL, USA) for 10 min to facilitate the solubilization of OLM. The obtained mixtures were then shaken for 72 h in an isothermal mechanical shaker (Clifton shaking water bath, London, UK) maintained at 40 °C to attain equilibrium. After reaching equilibrium, the equilibrated samples were centrifuged at 3000 rpm for 15 min to precipitate the undissolved OLM. Aliquots from the supernatants were then withdrawn and filtered through a membrane filter (0.45 µm, Whatmann, Maidstone, UK). Filtered solutions were suitably diluted with methanol and drug concentrations were determined using Hitachi UV–Vis spectrophotometer (Hitachi, Chiyoda, Japan) at λ_max_ 256 nm [17]. All measurements were done in triplicate and the solubility was expressed as the mean value (mg/mL) ± SD.

##### Preliminary Screening of Surfactants for Emulsification Efficiency

Different surfactants (Cremophor RH 40, Cremophor S9, Labrasol, Tween 20, Tween 40, Tween 60, Tween 80, Span 20 and Span 80) were screened for their emulsification ability in the selected oily phase. Surfactant selection was done on the basis of transparency percentage and ease of emulsification [18]. Briefly, 500 µL of each surfactant was added to 500 µL of the selected oil. The mixtures were gently heated at 50 °C for 2 min to attain homogenization. From each mixture, 100 µL were then diluted with distilled water up to 50 mL in glass stoppered flask. The stoppered flasks were inverted several times and the number of flask inversions required to form a homogenous nanoemulsion (with no turbidity or phase separation) was counted. Furthermore, the formed emulsions were allowed to stand for 2 h and their percentage transmittance was assessed at 650 nm (by means of UV–Vis Spectrophotometer) using distilled water as blank. The percentage transmittance was calculated for each emulsion in triplicate and the average values ± SD were calculated. The surfactant forming a clear emulsion with fewer inversions and higher percentage transmittance was selected [19].

##### Preliminary Screening of Cosurfactants for Emulsification Efficiency

The selected oily phase and surfactant were used for further screening of the different cosurfactants (Transcutol HP, PEG 200, PEG 400, PEG 600, Propylene glycol and Glycerin) for their emulsification efficiency. Mixtures of 200 µL of cosurfactant, 400 µL of selected surfactant and 600 µL of selected oil were prepared and evaluated in the same manner as described in preliminary screening of surfactants [20].

##### Construction of Pseudoternary Phase Diagram

In order to determine the concentration of components for the existing range of the SNEDDS, a pseudoternary phase diagram was constructed at ambient temperature using a water titration method [5]. Oil, surfactant and cosurfactant were grouped in different combinations for phase studies. Surfactant and cosurfactant (Smix) in each group were mixed in different weight ratio (1:0, 1:1, 1:2, 1:3, 2:1 and 3:1). These Smix ratios were chosen in increasing concentrations of surfactant with respect to cosurfactant and in increasing the concentration of cosurfactant, with respect to surfactant. For each phase diagram, the oil and specific Smix ratio were mixed thoroughly in different weight ratios (1:9, 1:7, 1:5, 1:4, 1:3, 1:2, 1:1 and 2:1) in different glass vials. Different ratios of oils and Smix were made to delineate the boundaries of each phase precisely [21]. The amount of aqueous phase was incremented by 5% to provide a concentration of aqueous phase in the range of 5%–95% of total volumes. After each addition of aqueous phase, the mixtures in the vials were vortexed for 2 min and allowed to equilibrate. The change in physical states from transparent to turbid and vice versa were visually observed and marked on the three component ternary phase diagram where each axis represented the oil, Smix and water, respectively. The different phase diagrams were plotted using CHEMIX ternary plot software (CHEMIX School Ver. 3.60, Pub. Arne Standnes).

#### 2.2.2. Preparation of OLM Loaded SNEDDS

Once the self-nanoemulsifying area was identified, SNEDDS formulae with desired component ratios were prepared. The ratio of surfactant to cosurfactant (Smix) was also optimized using pseudoternary phase diagrams. A series of SNEDDS formulae were prepared with varying weight ratios of selected oil (5%–15% *w*/*w*) and Smix (20%–80% *w*/*w*) as presented in Table 1. In all formulae, the amount of OLM was kept constant. Briefly oil, surfactant and cosurfactant were accurately weighed and mixed in stoppered glass vials using a vortex mixer to ensure complete mixing. An amount of OLM was dispersed into the mixture of oil and Smix with continuous mixing until OLM was completely dissolved. These systems were warmed to 40 °C using a water bath for 30 min with mild shaking until a clear solution was obtained. The prepared formulae were then stored at room temperature until further use [22].

#### 2.2.3. Characterization and Evaluation of OLM Loaded SNEDDS

##### Thermodynamic Stability Studies

The prepared SNEDDS formulae were subjected to heat-cool cycles, centrifugation and freeze-thaw cycles, where the physical appearances of the formulae were visually observed at the end of each stage. In heat-cool cycles, the prepared formulae were subjected to six cycles between 4 and 45 °C with storage at each temperature for 48 h. The formulae that did not show any phase separations, creaming or cracking were subjected to centrifugation at 3500 rpm for 30 min. Finally, only formulae which passed the previous two steps were stored at alternating temperatures of −21 and 25 °C, with the storage of 48 h at each temperature, for three cycles [23].

##### Robustness to Dilution

In order to simulate in vivo dilution behavior, effect of dilution on emulsion characteristics was studied. This test was performed by diluting 1 mL of each formula 10, 100 and 1000 times with distilled water, 0.1 N HCl and phosphate buffer pH 6.8. The diluted systems were mixed using a magnetic stirrer at 100 rpm and 37 °C to simulate body temperature to complete homogeneity. These systems were stored at an ambient temperature for 24 h then visually observed for any signs of phase separation [24].

##### Assessment of Efficiency of Self-Emulsification

The self-emulsification efficiency of SNEDDS was evaluated using a standard USP dissolution apparatus type II (Erweka, DT 600, Heusenstamm, Germany). 1 mL of each formula was added to 500 mL of distilled water maintained at 37 ± 0.5 °C. Gentle agitation was provided by a standard stainless steel dissolution paddle rotating at 50 rpm. The prepared formulae were assessed visually according to the rate of emulsification and final appearance of the nanoemulsion. The in vitro performance of the formulation was visually evaluated using the following grading system [25]:

Grade A: Rapidly forming emulsion having a clear or bluish appearance (within 1 min).

Grade B: Rapidly forming, slightly less clear emulsion, having a bluish white appearance.

Grade C: Fine milky emulsion that formed within 2 min.

Grade D: Dull, grayish white emulsion having slightly oily appearance that is slow to emulsify (longer than 2 min).

Grade E: Formula exhibiting either poor or minimal emulsification with large oil globules present on the surface.

##### Self-Emulsification Time

In this test, a predetermined volume of each formula (1 mL) was introduced into 300 mL of distilled water maintained at 37 ± 0.5 °C in a glass beaker and the contents were mixed gently using a magnetic stirrer rotating at constant speed (100 rpm). The emulsification time (the time required for a preconcentrate to form a homogeneous mixture upon dilution) was monitored by visually observing the disappearance of SNEDDS and the final appearance of the nanoemulsion [26].

##### Viscosity Determination

The viscosity of the prepared SNEDDS formulae was measured at 25 ± 0.5 °C as such before and after dilution by Brookfield viscometer (Brookfield Engineering Labs, Middleboro, MA, USA) using spindle CC3-14 with shear rate at 100 rpm [27].

##### Spectroscopic Characterization of Optical Clarity

The optical clarity of aqueous dispersions of SNEDDS formulae was measured spectrophotometrically. Composition was prepared according to the design and diluted to 100 times with distilled water. The percentage transmittance as a determinant of optical clarity for the prepared SNEDDS formulae was measured at 650 nm using distilled water as blank [28].

##### Transmission Electron Microscopy (TEM)

The surface morphology and globule size of the prepared SNEDDS formulae were observed using Transmission electron microscopy (JEM-2100, Pleasanton, CA, USA). Prior to analysis, the SNEDDS samples were diluted 10 times with distilled water. A drop from the resultant nanoemulsion was deposited on a film-coated copper grid forming a thin liquid film. The films were then negatively stained with 2% (*w*/*v*) phosphotungstic acid solution. After air drying, the stained films were photographed by transmission electron microscopy [29].

##### Droplet Size Analysis and Polydispersibility Index (PDI) Determination

The droplet size is an important factor in self-emulsification performance because it determines the rate and extent of drug release as well as absorption. Prior to measurement, 1 mL of each SNEDDS formula was diluted 10 times with distilled water. The globule size and polydispersibility index of the formed nanoemulsions were determined by dynamic light scattering (DLS) using a photon correlation spectrometer (Zetasizer, Malvern Instruments LTD, Malvern, UK) which analyzes the fluctuations in light scattering due to Brownian motion of the particles. Light scattering was monitored at 25 °C at a scattering angle of 90° [30]. All measurements were done in triplicate and the mean ± SD was calculated.

##### Zeta Potential Determination

The zeta potential of the diluted SNEDDS formulae was determined using Zetasizer (Malvern Instruments). Samples were placed in clear disposable cuvette and results were recorded. Charge on emulsion droplets and their zeta potential values were obtained [30].

##### Drug Loading Efficiency

For determining the OLM content, 1 mL of SNEDDS formulae (equivalent to 20 mg of OLM) was diluted with methanol in volumetric flask (VF) and mixed well by shaking or inverting the VF two to three times. Samples were prepared in triplicate and absorbance was measured after suitable dilutions at 256 nm using UV–Vis Spectrophotometer (Hitachi U-2900, Tokyo, Japan). The amount of OLM present in each formula was calculated from a calibration plot [31].

##### In Vitro Drug Release Studies

The in vitro drug release of OLM from the optimized SNEDDS formulae, pure drug and marketed product was performed using USP dissolution apparatus type II (Erweka, DT 600). The dissolution medium consisted of 900 mL of freshly prepared phosphate buffer pH 6.8 maintained at 37 ± 0.5 °C and the paddle speed was set at 50 rpm. Hard gelatin capsules, size “000” filled with preconcentrate (equivalent to 20 mg Olmesartan) were tied to paddles using para film spring to prevent capsules from floating. Aliquots (5 mL) from the dissolution medium were withdrawn at regular time intervals (5, 10, 15, 30, 45, 60, 90 and 120 min) using a calibrated disposable syringe. The withdrawn samples were replaced by equal volumes of dissolution medium to maintain the volume and sink conditions constant. The samples were then filtered through a membrane filter (0.45 µm, Whatmann) and drug concentration was obtained after proper dilutions via UV validated method at 250 nm using UV–Vis Spectrophotometer (Hitachi U-2900). All measurements were done in triplicate [32].

##### Kinetic Treatment for the in Vitro Release of OLM SNEDDS (Release Kinetic Modeling)

Kinetic treatment is a necessary step for the evaluation of in vitro release of different formulae. Furthermore, the in vitro release and its kinetic treatment are often used to predict the way by which the drug in such a system may perform in vivo. The in vitro release data were analyzed using different kinetic models and the important parameters of each order were calculated [33]. For all prepared OLM formulae, five parameters were determined and calculated in the applied orders and models. These parameters are intercept, slope of the dissolution curve, correlation coefficient (*r*), reaction rate constant (K) and half-life (*t*_1/2_).

#### 2.2.4. Preparation of OLM Loaded S-SNEDDS

Based on the rank order performed for all conventional OLM SNEDDS formulae depending on their characterization and evaluation tests, two optimized SNEDDS formulae were selected to be solidified by spray drying technique using Aerosil 200 as solid carrier. Briefly, SNEDDS formula and Aerosil 200 (1000 mg) were suspended in 200 mL ethanol with continuous stirring until forming an isotropic mixture. The mixture was then kept at room temperature and equilibrated for 24 h. The suspension was then spray dried using a Buchi mini spray dryer (Buchi, B-190, Basel, Switzerland) under the following conditions: inlet temperature, 60 °C; outlet temperature, 35 °C; aspiration, 85%; feeding rate of the suspension, 5 mL/min and atomization air pressure, 5 kPa [34].

#### 2.2.5. Characterization of OLM Loaded S-SNEDDS

##### Micromeritic Properties of S-SNEDDS

*Angle of Repose (θ)*. The angle of repose of S-SNEDDS was determined by funnel method. Accurately weighed samples were taken in a funnel. Height of the funnel was adjusted in such a way that the tip of the funnel just touched the apex of the heap of S-SNEDDS powder. The powder was allowed to flow freely through the funnel onto the surface. The diameter of the powder cone was measured and angle of repose calculated using the following equation [35]:

tan θ = *h*/*r*

where, *h* = height of the heap, *r* = radius of the heap.

*Bulk and Tapped Density*. Both bulk density (BD) and tapped density (TD) were determined. A quantity of 2 g of S-SNEDDS was introduced into a 10 mL measuring cylinder. Initial volume was observed, then the cylinder was allowed to fall under its own weight onto a hard surface from a height of 2.5 cm at 2 s intervals. The tapping was continued until no further change in volume was noted (approximately 5 tappings until constant volume). Bulk density and tapped density were calculated using the following equations [36]:

BD = Weight of powder/Bulk Volume

TD = Weight of powder/Tapped Volume

*Compressibility Index*. The compressibility of the S-SNEDDS granules was determined by Carr’s Compressibility Index as follow [37]:

Carr’s Compressibility Index (%) = [(TD − BD)/TD] × 100

*Hausner Ratio*. It is the ratio of tapped density to bulk density. It gives an idea about the flow characters of powder particles and can be calculated as follow [36]:

Hausner ratio = TD/BD

*Reconstitution Properties of S-SNEDDS*. Reconstituted S-SNEDDS were characterized for robustness to dilution, self-emulsification time, droplet size analysis and PDI as described for liquid SNEDDS in Section 2.2.3.

*Scanning Electron Microscopy (SEM)*. Scanning electron micrographs for OLM, Aerosil 200 and prepared S-SNEDDS formulae were taken using Scanning electron microscope (JEOL, JSM 50A, Tokyo, Japan) operating at 20 kV to study surface topography of S-SNEDDS. The samples were fixed on SEM stub and then coated with thin layer of platinum [37].

*Differential Scanning Calorimetry (DSC)*. Physical state of OLM in S-SNEDDS was characterized using differential scanning calorimeter. Thermograms of OLM, Aerosil 200, physical mixture of both and prepared optimized S-SNEDDS formulae were obtained using differential scanning calorimeter (Shimadzu, DSC-50, Kyoto, Japan). The thermal behavior was studied by heating nearly 2 mg of samples in sealed aluminum pans under nitrogen gas flow (30 mL/min) over a temperature range of 0 to 250 °C and a heating rate of 10 °C/min [38].

*Fourier Transformed Infrared Spectroscopy (FTIR)*. FTIR Spectra of pure OLM, Aerosil 200, physical mixture of both and prepared optimized S-SNEDDS formulae were obtained using Fourier transformed infrared spectrophotometer (Shimadzu 8400). Solid samples were mixed with small quantity of IR grade potassium bromide and compressed into discs by applying pressure. The compressed disc was placed in light path and the spectrum was obtained. Each KBr disc was scanned at 4 mm/s at a resolution of 2 cm over a wave number region of 4000–400 cm^−1^ [39].

*Drug Loading Efficiency*. OLM content in S-SNEDDS formulae was estimated using the method previously mentioned for liquid SNEDDS in Section 2.2.3 [31].

*In Vitro Drug Release Studies*. The in vitro drug release of OLM from the optimized S-SNEDDS formulae was performed using the above mentioned method for conventional liquid SNEDDS in Section 2.2.3 [32].

*Pharmacokinetic Study*. Male Wister rats (weighing approximately 250 ± 30 g) were used for the bioavailability study. The animals were housed three per cage in the laboratory with free access to food and water and maintained on a 12 h dark/light cycle in a room with controlled temperature (25 ± 1 °C) and humidity (55% ± 5%). All the procedures used in the present study were conducted according to the guidelines approved by the Institutional Animal Care and Ethical Committee of Suez Canal University (18 February 2013). The rats were randomly divided into three groups (*n* = 6) and deprived of food but had free access to water 24 h before the day of experiment. They were treated orally by oral gavage using an animal feeding needle as follow [37]:

Group I: Pure drug in 0.5% sodium carboxy methylcellulose as suspending agent.

Group II: Marketed conventional tablet suspended in 0.5% sodium carboxy methylcellulose (Reference).

Group III: Optimized S-SNEDDS formula redispersed in 1 mL distilled water (Test).

Blood samples (0.5 mL) were collected via the retro-orbital vein at predetermined time points (0, 1, 2, 3, 4, 8, 12 and 24 h) after oral administration into heparinized microcentrifuge tubes. The blood samples were immediately centrifuged at 5000 rpm for 10 min to separate plasma. The plasma samples were collected and stored at −20 °C until drug analysis [40]. Frozen plasma samples were thawed at room temperature. The plasma samples (100 µL) were separated and 0.9 mL of Acetonitrile was added to each of plasma samples to precipitate the protein. The samples were then centrifuged again at 5000 rpm for 5 min and the supernatant (20 µL) were filtered and directly injected into the HPLC column and peak area values were recorded. The HPLC instrument used was equipped with a model series L-2000 organizer box, L-2300 column oven, L-2130 pump with built in degasser, Rheodyne 7725i injector with a 20 µL loop and L-2455 diode array detector (DAD). Separation and quantitation were made on a 250 mm × 4.6 mm (i.d.), 5 µm particle size ODS reverse-phase column kept at room temperature. The mobile phase for analysis of OLM in plasma consisted of methanol and phosphate buffer pH 4.5 with a ratio of 70:30 at a flow rate of 1 mL/min. The column temperature was kept at 25 °C. The plasma concentrations vs. time profiles were analyzed using pharmacokinetic software (PK function for Microsoft Excel, Pharsight Corporation, Sunnyvale, CA, USA) adopting non-compartmental analysis. Data from the plasma concentration time curve within 24 h after drug intake were employed to estimate the following pharmacokinetic parameters for individual rats in each group, peak plasma concentration (*C*_max_), time to reach peak plasma concentration (*t*_max_), area under the plasma concentration vs. time curve from zero to the last sampling time (AUC_0–24h_) and half life (*t*_1/2_) for both IRB and OLM [41].

## 3. Results and Discussion

### 3.1. Preformulation Study (Selection of SNEDDS Components)

#### 3.1.1. Study of OLM Solubility in Various Oils, Surfactants and Cosurfactants

**Figure 2:**
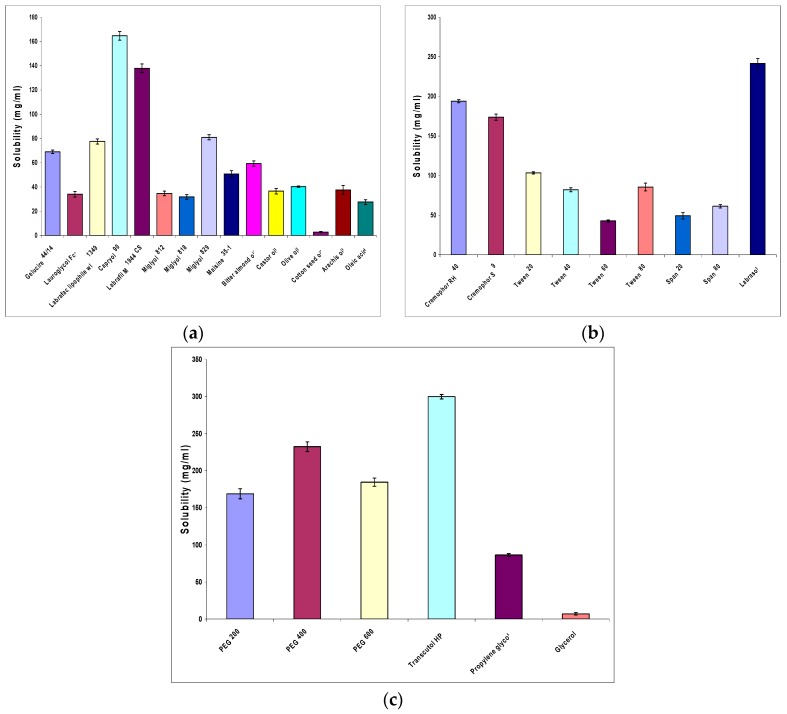
Solubility of OLM (**a**) in various oils; (**b**) in various surfactants; (**c**) cosurfactants.

To design a SNEDDS with acceptable physicochemical characteristics, the components of the system including oil, surfactant and cosurfactant must be carefully chosen. Solubility studies aimed at identifying suitable SNEDDS components with good solubilizing capacity for OLM. Identifying the suitable oil, surfactant and cosurfactant having maximal solubilizing potential for drug under investigation is very important to achieve optimum drug loading [42]. Oils can solubilize the lipophilic drug in a specific amount so they are the main excipients because they can increase the fraction of lipophilic drug transported via the intestinal lymphatic system, thereby increasing absorption from the GIT. Capryol 90 was selected as an oily phase for OLM due to its highest solubilization (164.69 ± 3.59 mg/mL) compared to other screened oils as shown in Figure 2a. This may be attributed to the medium chain length (eight carbons) and the amphiphilic nature of Capryol 90 which provide it with surfactant properties and therefore, enhanced drug solubilization, as explained by Balakrishnan et al. [28]. Besides its high drug solubilization power, Capryol 90 being a saturated medium chain fatty acid with HLB value equal 6, is known for its efficient self-emulsification properties which aid the formation of the self-emulsifying system containing the drug [43]. All the investigated surfactants in this study were non-ionic hydrophilic ones. Being non-ionic, the investigated surfactants are considered safe and biocompatible [44] and being hydrophilic (with HLB values >10), they are superior in forming fine, uniform emulsion droplets which can empty rapidly from the stomach and provide a large surface area that facilitates rapid drug release and absorption. Surfactants also form a layer around the emulsion droplets and hence reduce the interfacial energy, as well as providing a mechanical barrier to coalescence. This can prevent precipitation of the drug within the GI lumen. Among the various surfactants screened, Labrasol showed the best solubilizing potential for OLM (241.51 ± 6.28 mg/mL) as illustrated in Figure 2b. Transient negative interfacial tension and a fluid interfacial film are rarely achieved with the use of a single surfactant, usually necessitating the addition of a cosurfactant. The presence of cosurfactants decreases the bending stress of the interface and allows an interfacial film with sufficient flexibility to assume different curvatures required to form a nanoemulsion over a wide range of compositions. Among the different cosurfactants used in this study, Transcutol HP exhibited maximum solubility for OLM (299.96 ± 2.98 mg/mL) as presented in Figure 2c.

#### 3.1.2. Preliminary Screening of Surfactants for Emulsification Efficiency

Although being a major parameter in choosing the ingredients of SNEDDS, drug solubility is not the only parameter governing the choice of the surfactant in the prepared systems. The emulsifying efficiency of the surfactant is rather a much more important factor [45]. Therefore, the emulsifying efficiency of different surfactants was screened regarding the selected oil. The ability of the surfactant to form an emulsion was assessed by the number of flask inversions needed for emulsion formation, while the stability of the formed emulsion was expressed by its percentage UV transmittance, two hours after preparation. Optical clarity corresponds to high transmittance, as opalescent dispersions will scatter incident radiation to larger extent as compared to transparent dispersions. The intensity of light passing through such dispersion is attributed to the scattering of light which occurs due to absence of optical homogeneities in the medium [46]. Hence, percentage transmittance could directly be used to predict relative droplet size of the emulsion. Based on this principle, aqueous dispersions with high transmittance (lower absorbance) were considered optically clear and oil droplets were thought to be in a state of nanodispersion [47]. The percentage transmittance values and number of flask inversions of various dispersions are listed in Table 2. For all screened surfactants, large number of flask inversions (7 inversions) was reported for Labrasol, indicating the most difficulty in emulsion formation. In addition, emulsions formed by Labrasol had the least stability as indicated by the least percent UV transmission reported (44.87% ± 0.95%). On the other hand, relatively few numbers of flask inversions (4 inversions) were needed for emulsion formation using Cremophor RH40 as emulsifying agents. Moreover, the percentage of UV transmission of the formed emulsions (two hours after preparation) approached 100% indicating an accepted stability of the formed emulsions. Thus, Cremophor RH40 was chosen as a surfactant for further investigation due to its better nanoemulsification efficiency. These observed differences in the emulsification efficiency of the investigated surfactants were attributed to the difference in their chain length and structure as explained by Lawrence in his study on microemulsions as drug delivery vehicles [48].

#### 3.1.3. Preliminary Screening of Cosurfactants for Emulsification Efficiency

All cosurfactants employed in this study appeared to improve the emulsification ability of Capryol 90 and Cremophor RH40. Addition of cosurfactant to the surfactant-containing formulation was reported to improve the dispersibility and drug absorption from formulation [49]. As depicted in Table 3, Transcutol HP exhibited good emulsification efficiency with Capryol 90 and Cremophor RH40 mixture, showing maximum transmittance (99.83% ± 0.06%) and 3 inversions only, compared to other employed cosurfactants.

#### 3.1.4. Construction of Pseudoternary Phase Diagram

**Figure 3:**
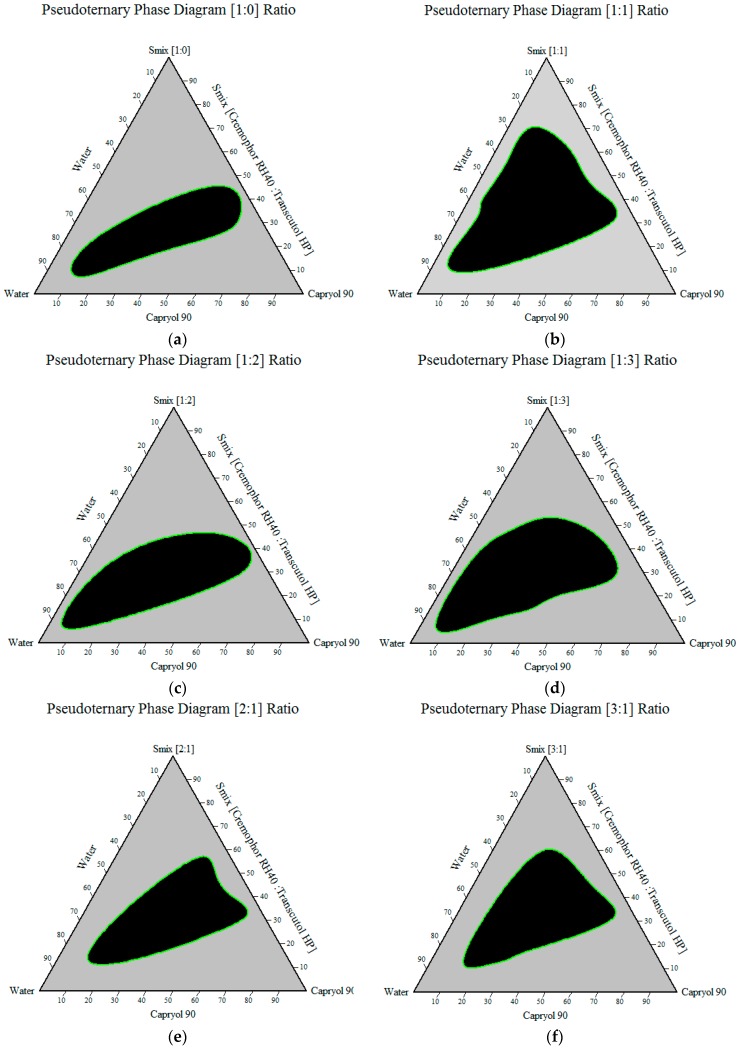
Pseudo-ternary phase diagram of Smix (**a**) [1:0]; (**b**) [1:1]; (**c**) [1:2]; (**d**) [1:3]; (**e**) [2:1]; (**f**) [3:1].

One of the most important characteristics of SNEDDS is the change that occurs when the system is diluted (since it will be diluted by body fluids after administration), which may cause drug precipitation due to the loss of solvent capacity [50]. Therefore, Pseudoternary phase diagrams were constructed to identify self-nanoemulsifying regions and to select suitable concentrations of oil, surfactant and cosurfactant for the formulation of SNEDDS. The phase diagrams were mapped at surfactant/cosurfactant ratios (1:0, 1:1, 1:2, 1:3, 2:1 and 3:1). The size of the nanoemulsion region in the diagrams was compared, the larger the size the greater the self-nanoemulsification efficiency. The nanoemulsion phase was identified as the area where clear and transparent formulae were obtained on dilutions based on visual inspection of samples. Pseudoternary phase diagrams showed that the zone of nanoemulsion (the black area) was largest in formulae prepared with Cremophor RH40-Transcutol HP mixture (Smix) at 1:1 ratio as shown in Figure 3. Thus, fixing the surfactant/cosurfactant ratio at 1:1 is a better choice from a stability point of view [51]. At Smix 1:1, and when cosurfactant was added with surfactant in equal amounts, a higher nanoemulsion region was observed, perhaps because of the further reduction of the interfacial tension and increased fluidity of the interface at Smix 1:1.

### 3.2. Characterization and Evaluation of OLM Loaded SNEDDS

#### 3.2.1. Thermodynamic Stability Studies

All OLM loaded SNEDDS formulae showed no signs of precipitation, cloudiness or separation after the heat-cool cycles, centrifugation and freeze-thaw cycles which ensured the stability of all reconstituted nanoemulsion. Visual observation indicated that there was no phase separation or any flocculation in all formulae and the physical appearance of all formulae was similar as shown in Table 4. Thus, the overall stability of all SNEDDS formulae under these stress conditions was found to be acceptable. These results concur with Ashok et al., who prepared Albendazole self-emulsifying drug delivery system (SEDDS) and found that a sudden change in temperature had no effect in the entropy of the system [52].

#### 3.2.2. Robustness to Dilution

The ability of SNEDDS to be diluted without any phase separation and drug precipitation is essential for its use as a drug delivery vehicle. After dilution of all SNEDDS formulae, the resulting nanoemulsions were found to remain clear, transparent and showed no phase separation even after 24 h as shown in Table 5. This gave a good indication about the suitability of such systems for oral application where they stood a greater chance of passing along the gastrointestinal tract as emulsified oil globules without phase separation. This implied that all OLM SNEDDS formulae were stable at infinite aqueous dilution. In addition, the composition and pH of the aqueous phase was found to have no effect on the properties of nanoemulsions [53]. Similar results were reported by Anuradha et al. who formulated Peppermint oil based drug delivery system of aceclofenac and found that after dilution, the resulting microemulsions were found to remain clear, transparent and appeared like homogenous single-phase liquids [46].

#### 3.2.3. Assessment of Efficiency of Self-Emulsification (Dispersibility Test)

The in vitro performances of the formulae were visually assessed using the grading system previously mentioned and the results are shown in Table 6. Visual observations showed that all SNEDDS formulae were found to be grade A. This ability of efficient self-emulsification is very important for SNEDDS as the emulsification process is considered the rate limiting process for drug absorption. Similar observations were reported by Naseem et al. who prepared self-nanoemulsifying lipid carrier system of Etoposide and found that six formulations were robust enough to exist in nanoemulsion form upon dispersion in the simulated aqueous environments of the GI tract and categorized them as grade (A) [41].

#### 3.2.4. Self-Emulsification Time

The efficiency of SNEDDS could be estimated primarily by determining the rate of emulsification. The recorded self-emulsification times for the tested OLM loaded formulae are represented in Table 7. From the results obtained, it was evident that all the tested formulae of OLM SNEDDS were self-emulsified within 14.10 ± 0.75 to 22.38 ± 0.77 s. The short self-emulsification time reported for all the investigated systems indicate their ability for easy and rapid emulsification. The results revealed that self-emulsification time depends mainly upon the individual composition and its proportion of oil, surfactant and cosurfactant. From the self-emulsification time results, it was remarked that as the concentration of surfactant increases, the spontaneity of emulsification process increased and self-emulsification time decreased. This may be due to the capacity of Cremophor RH40 in reducing the interfacial tension and thus excess diffusion of the aqueous phase into the oil occurs, causing significant interfacial disruption and discharge of droplets into the bulk aqueous phase [54]. These results concur with those of Shailesh et al. who prepared Self-nanoemulsifying drug delivery system (SNEDDS) of Olmesartan medoxomil and found that all prepared SNEDDS formulae showed far less emulsification time in the range of 15 to 35 s [40].

#### 3.2.5. Viscosity Determination

**Figure 4:**
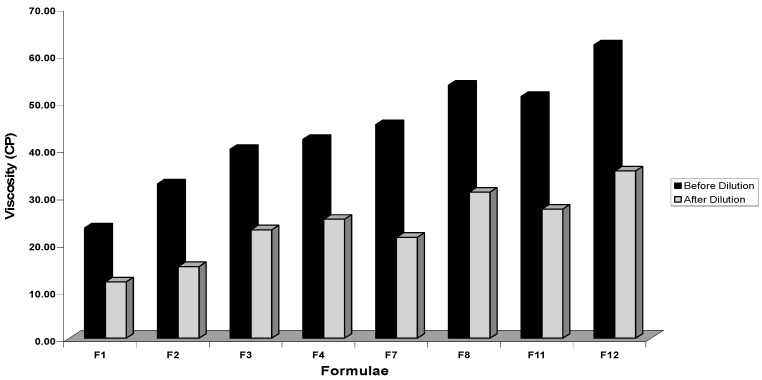
Plot of viscosity before and after dilution for various OLM SNEDDS formulae.

Viscosity studies are necessary for SNEDDS to characterize the system physically and to control its stability. The viscosity of SNEDDS is critical during its dispersion in the aqueous phase. Higher viscosities tend to slow down the emulsification rate which may affect in vivo drug release and bioavailability profiles. From viscosity determination results, it was observed that as the concentration of oil and Smix increased, the viscosity of SNEDDS formulae also was increased as shown in Figure 4. The OLM SNEDDS formulae had the average viscosity range between 23.35 ± 0.84 and 62.15 ± 1.69 cps. However, after dilution with 100 times distilled water the viscosity range decreased and ranged between 11.94 ± 1.39 and 35.41 ± 0.74 cps. All formulae were found to have rather low viscosities which indicated the resulted nanoemulsion to be O/W type. The viscosity values recorded by the SNEDDS formulae in the present study were low enough to preclude the possibility of rapid self-emulsification [55].

#### 3.2.6. Spectroscopic Characterization of Optical Clarity

The percentage transmittance is an important index to determine the isotropic nature of SNEDDS. A value of percent transmittance closer to 100% signified that all selected formulae were clear and transparent. Besides signifying clarity of the formulation, a percentage transmittance closer to 100% also implied that the size of the globules approximated the nanometric range, which in turn indicated that the formula had a large surface area for drug release, high capacity to undergo enhanced absorption in biological matrix and thus had the ability for increased oral bioavailability [56]. On 100 fold dilution, the percentage transmittance of OLM SNEDDS formulae was found to be between 97.97% ± 0.32% and 99.60% ± 0.14% as shown in Table 7. The above results of percentage transmittance confirm the good transparent nature of all OLM loaded SNEDDS formulae. These results are in alignment with the results reported by Maulik et al. who prepared stable SMEDDS of lovastatin and found that the percentage transmittance of the prepared SNEDDS formulae were close to 100% [57].

#### 3.2.7. Transmission Electron Microscopy (TEM)

**Figure 5:**
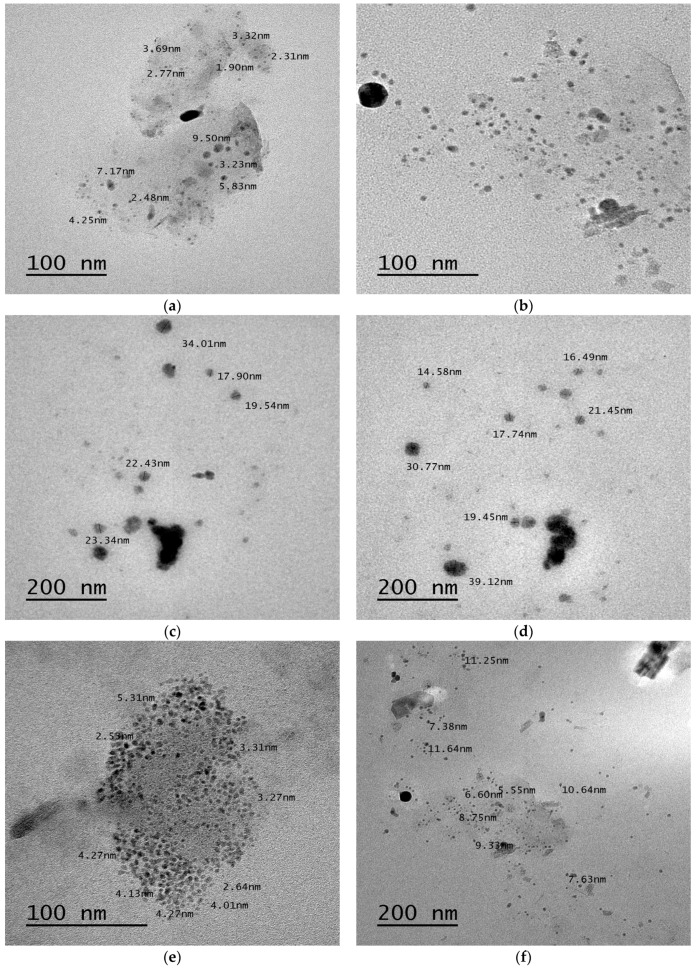

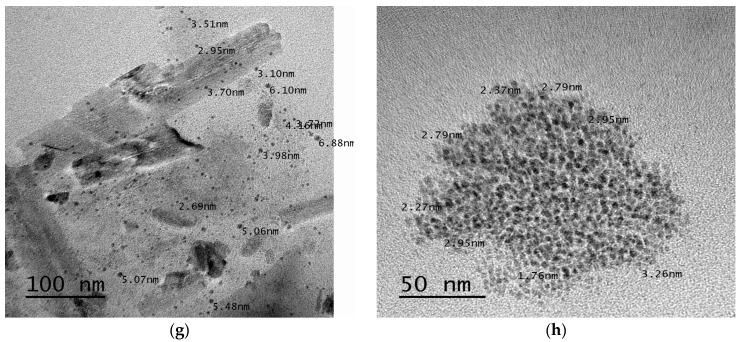
TEM photograph of F1–F8.

TEM photographs of OLM loaded SNEDDS formulae subsequent to post dilution with distilled water are shown in Figure 5 and interpreted for surface morphology and globule size. From the presented figures, it was apparent that globules of all formulae were well dispersed and no globule aggregation took place. TEM analysis revealed that most formulae showed spherical and homogeneous droplets with a size smaller than 50 nm, which satisfies the criteria of nanometric size range required for nanoemulsifying formulae [58].

#### 3.2.8. Droplet Size Analysis and Polydispersibility Index (PDI) Determination

The droplet size is the crucial factor in the SNEDDS performance because it determines the rate and extent of drug release as well as drug absorption. Moreover, it has been reported that the smaller the particle size, the larger the interfacial surface area which may lead to more rapid absorption and improve the bioavailability. Systems with a mean droplet size below 200 nm fulfill the criteria of SNEDDS. All the investigated systems had a mean globule size of less than 50 nm indicating their efficiency as SNEDDS. The small globule size of the diluted systems can be attributed to the use of the proper surfactant/cosurfactant mixture. This provided adequate reduction in the free energy of the system which in turn resisted the thermodynamic instabilities on changing the environment pH and volume. Also, the surfactant/cosurfactant mixture provided a strong mechanical barrier protecting the formed globules from being aggregated as explained by Nepal et al. [59]. From droplet size analysis it was observed that OLM loaded SNEDDS formulae had the mean particle size in the range of 14.91 ± 0.12 to 22.97 ± 0.44 nm indicating their efficiency as SNEDDS as presented in Table 7. It was also noticed that as the surfactant percentage increased, the mean droplet size decreased [60]. The decrease in droplet size may be due to more surfactant being available for adsorption and the formation of a more closely packed surfactant film at the oil-water interface, thereby providing a stable and condense interfacial film, as well as the low interfacial tension in the system. The mean droplet size is not the only parameter to be considered in the formulation of SNEDDS. The droplet size distribution is another parameter of equal importance. The droplet size distribution is expressed by a dimensionless value called the polydispersibility index (PDI) which is the measure of particle homogeneity and it varies from 0.0 to 1.0. The closer to zero the PDI value, the more homogenous are the particles. The small values of PDI shown by all SNEDDS formulae (0.036 ± 0.05–0.241 ± 0.09) indicate a homogenous droplet population and narrow globule size distribution as reported in Table 7. This in turn indicates more uniform emulsions with higher physical stability.

#### 3.2.9. Zeta Potential Determination

The magnitude of the zeta potential gives an indication of the potential stability of the colloidal system. If all the particles have a large negative or positive zeta potential, they will repel each other creating dispersion stability. If the particles have low zeta potential values, then there is no force to prevent the particles coming together and there is dispersion instability. A dividing line between stable and unstable aqueous dispersions is generally taken at either +30 or −30 mV. Particles with zeta potentials more positive than +30 mV are normally considered stable. Particles with zeta potentials more negative than −30 mV are normally considered stable [24]. The zeta potential values of OLM loaded SNEDDS formulae were in the range of −2.63 ± 0.47 to −5.13 ± 0.61 mV as shown in Table 7. It was observed that the absolute values of zeta potential were lower than those values reported in the literature. This might be attributed to the presence of Cremophor RH40 and Transcutol HP, non-ionic surfactants which sterically stabilize the system by forming a coat around their surface. The non-ionic surfactants do not contribute any charge to the nanoemulsion particle and this indicates that negative charge particles do not affect the stability of formed nanoemulsion [61]. Our results also agreed with Kulkarni et al. who prepared solid self-nanoemulsifying formulation of Rosuvastatin Calcium and found that the zeta potential values were negative in between −4.93 and −11.8 mV [62].

#### 3.2.10. Drug Loading Efficiency

The drug loading efficiency for all OLM SNEDDS formulae was found in the range of 92.37% ± 0.75% (F1) to 99.09% ± 0.56% (F8), indicating uniform drug dispersion in formulae as presented in Table 7. Statistically it was further justified that there was no significant difference in drug content among the various formulae. It was observed that formula F8 have the highest drug content. This may be attributed due to higher concentration of surfactant and cosurfactant in these two formulae that possess high solubilizing capacity to solubilize the 20 mg dose of OLM. These observations are in line with studies reported by Ashish et al. who found that the drug content of Furosemide SNEDDS in between 90.08% ± 0.124% and 102.45% ± 0.312% [63].

#### 3.2.11. In Vitro Drug Release Studies

**Figure 6:**
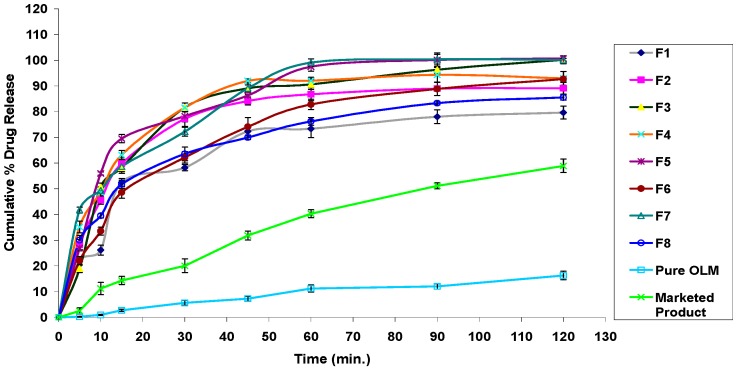
In vitro release profiles of OLM SNEDDS formulae compared with pure OLM and marketed product.

The in vitro release profiles of different OLM SNEDDS formulae together with the release profile of pure OLM filled in hard gelatin capsules and that of OLM marketed tablets are presented in Figure 6. Also, no dissolution-accelerating components or surfactants such as sodium lauryl sulfate were added to the media as these components result in failure to discriminate between different in vitro release profiles, as the surfactant is the key element in improving dissolution of SNEDDS dosage forms [64]. Figure 6 signifies that in vitro release profiles of OLM from SNEDDS formulae produced a constantly superior drug release rate as compared to that of pure drug and marketed tablets. Within the initial one hour of the in vitro release study, only 11.23% ± 1.42% and 40.33% ± 1.56% of OLM was dissolved from pure drug and marketed tablets respectively, whereas the SNEDDS formulae showed improved release within the same time period where more than 90% of OLM was released from formulae F3, F4, F5 and F7. OLM dissolved and released from SNEDDS reached 90.66% ± 2.08% for formula F3, 92.08% ± 1.33% for formula F4, 97.51% ± 1.72% for formula F5 and 99.11 ± 1.44 for formula F7 within the initial one hour. This enhancement in OLM in vitro release rate and extent could be attributed to the spontaneous formation of nanoemulsion during dissolution process with droplet size in the nanometric range. This induced the presentation of OLM at a dissolved state in the form of nanoemulsion which leads to an increased solubilization and enhanced drug dissolution rate and extent. Thus, this greater availability of dissolved OLM from the SNEDDS formulae could lead to higher absorption and higher oral bioavailability. In contrast to this, the release of OLM from formulae F6 and F8 were significantly lower than from other formulae, which may be attributed to the higher percentage of surfactants employed in these two formulae resulting in higher probability of surfactant migration into surrounding aqueous media upon dispersion. This process leads to formation of micelles that traps the free drug creating hindrances in drug release [65]. It was also observed that there was a good correlation between the droplet size of generated nanoemulsions after reconstitution of SNEDDS and the in vitro release of OLM. In another words, the amount of drug dissolved in the aqueous phase at time *t*, is inversely proportional to the droplet size of the generated nanoemulsions after SNEDDS reconstitution [21]. Thus, this rapid drug release was promoted by the larger interfacial areas present in emulsions with smaller drops [13].

#### 3.2.12. Kinetic Treatment for the in Vitro Release of OLM SNEDDS (Release Kinetic Modeling)

The best kinetic order for the in vitro release of OLM from SNEDDS formulae can be calculated from the highest values of the obtained correlation coefficients (*r*). Table 8 showed that the in vitro release of OLM from all SNEDDS formulae obeys Higuchi’s diffusion model. On modeling, the diffusion of OLM from SNEDDS formulae exhibited higher correlation coefficients values for the Higuchi model compared with other applied orders and models. This may be due to the rapid diffusion OLM from diffusion system (SNEDDS) used which has a reservoir compartment (donor compartment). Also, the presence of insoluble ingredient within the formulae helps to keep up the physical dimension of hydrophobic matrix throughout drug release. As such, diffusion of active ingredient from the system is that the release mechanism and therefore the corresponding release characteristic is represented by Higuchi equation.

### 3.3. Preparation of OLM Loaded S-SNEDDS

Based on the rank order performed for all conventional OLM SNEDDS formulae depending on their characterization and evaluation tests, two optimized SNEDDS formulae were selected to be converted into S-SNEDDS. From the in vitro drug release data, drug loading efficiency and particle size analysis formulae F3 and F4 were selected as optimized formulae to be solidified into S-SNEDDS as illustrated in Table 9.

### 3.4. Characterization of OLM Loaded S-SNEDDS

#### 3.4.1. Micromeritic Properties of S-SNEDDS

The values obtained for the angle of repose of the two S-SNEDDS formulae F3 and F4 were 27.64° ± 1.03° and 22.79° ± 2.22° respectively, as shown in Table 10. These values indicate that all formulae have good flowability. The bulk density of the two formulae F3 and F4 was found to be 0.49 ± 0.02 g/mL and 0.51 ± 0.03 g/mL respectively. However, tapped density was 0.59 ± 0.02 g/mL for formula F3 and 0.54 ± 0.01 g/mL for formula F4. Carr’s index of formulae F3 and F4 was found to be 14.32 ± 1.72 and 12.41 ± 0.95 respectively which give an indication about the good flowability of the two S-SNEDDS formulae. This was further supported by the values of Hausner’s ratio. The results of Hausner ratio of formulae F3 and F4 were 1.22 ± 0.10 and 1.08 ± 0.08 respectively. The improved flowability of S-SNEDDS formulae may be due to good sphericity of particles.

#### 3.4.2. Reconstitution Properties of S-SNEDDS

A dilution study was done to observe the effect of dilution on S-SNEDDS, because dilution may better mimic the condition of stomach after oral administration. It was observed that the two S-SNEDDS formulae F3 and F4 disperse quickly and completely when subjected to an aqueous environment under mild agitation. The two formulae showed spontaneous nanoemulsification and there was no sign of phase separation or phase inversion of nanoemulsion after storage of 24 h. The efficiency of self-emulsification can also be estimated by measuring the emulsification time. The emulsification time was 43.51 ± 1.29 s. for formula F3 and 35.68 ± 0.91 s. for formula F4. It was also observed that reconstituted nanoemulsion of formula F3 had the mean particle size 75.92 ± 2.34 while formula F4 had the particle size 32.22 ± 1.95. The small values of PDI shown by S-SNEDDS formulae F3 (0.407 ± 0.14) and F4 (0.315 ± 0.27) indicate homogenous droplet population and narrow globule size distribution. It was also noticed that the emulsification times, droplet size and PDI for liquid SNEDDS and S-SNEDDS were very close to each other, indicating that the spray-drying process did not have a remarkable influence on the emulsfication performance of S-SNEDDS. These results were in complete accordance with Chun Chao et al. who prepared solid lipid-based self-emulsifying drug delivery system of agaricoglycerides and found that the spray-drying had no effect on the emulsfication performance [66].

#### 3.4.3. Scanning Electron Microscopy (SEM)

**Figure 7:**
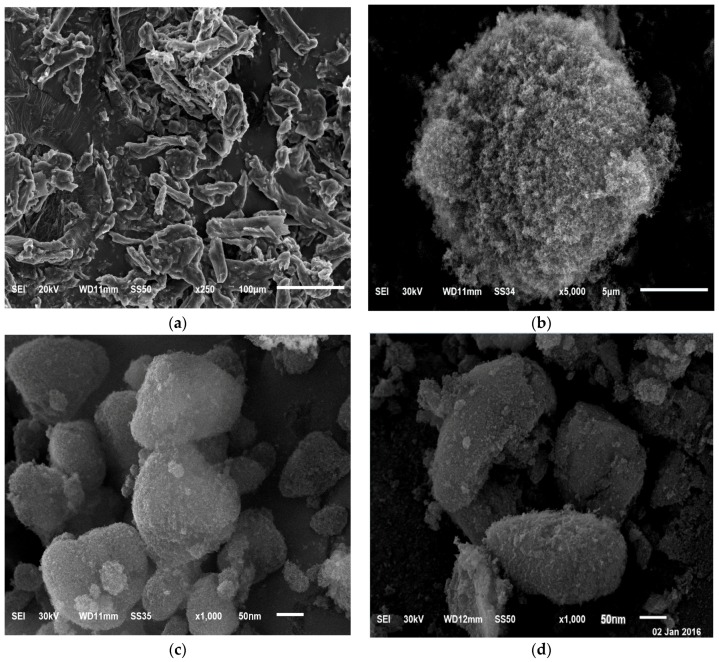
SEM photograph of pure (**a**) OLM; (**b**) Aerosil 200; (**c**) S-SNEDDS F3; (**d**) S-SNEDDS F4.

The surface morphology of pure OLM powder, Aerosil 200 and S-SNEDDS formulae of OLM was determined using scanning electron microscope as shown in Figure 7. The OLM powder appeared with an irregular crystalline shape as irregular and plate-shaped crystals having rough surfaces. Aerosil 200 appears to be spherical porous particles. The image of the solid SNEDDS formulae (F3 and F4) containing OLM however, illustrate that the particles had the same outer macroscopic morphology consisting of well separated spherical particles with relatively deep dents and similar diameters. Following spray drying, the crystalline OLM turned out to be highly amorphous in nature.

#### 3.4.4. Differential Scanning Calorimetry (DSC)

**Figure 8:**
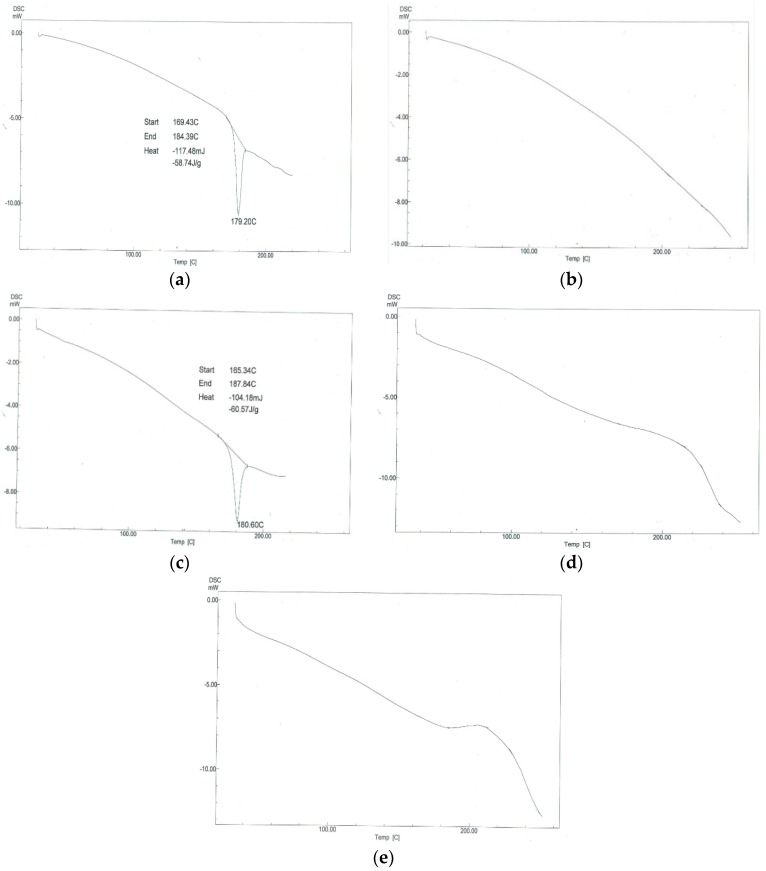
DSC thermograms of (**a**) pure OLM; (**b**) Aerosil 200; (**c**) physical mixture of OLM and Aerosil 200; (**d**) OLM S-SNEDDS formula (F3); (**e**) OLM S-SNEDDS formula (F4).

Thermograms of pure OLM, Aerosil 200, physical mixture of both and prepared optimized S-SNEDDS formulae (F3 and F4) were obtained using differential scanning calorimeter as shown in Figure 8. The thermogram of pure OLM exhibited a sharp endothermic peak at about 179.20 °C, corresponding to its melting point as shown in Figure 8a. Aerosil 200 showed no specific peaks from 0 to 250 °C as presented in Figure 8b. However, a melting endotherm having the characteristic peak of OLM was observed in the physical mixture of OLM and Aerosil 200. In case of OLM S-SNEDDS formulae (F3 and F4), the endothermic peak of OLM was absent as shown in Figure 8d,e. The change in melting behavior of OLM can be attributed to the inhibition of its crystallization and solubilization of OLM in S-SNEDDS. Therefore, it could be concluded that OLM in the solid SNEDDS was in the amorphous form. It is known that transforming the physical state of a drug to the amorphous or partially amorphous state leads to a high-energy state and high disorder, resulting in enhanced solubility [34]. As a result, it was expected that the solid particles would also have enhanced solubility.

#### 3.4.5. Fourier Transformed Infrared Spectroscopy (FTIR)

**Figure 9:**
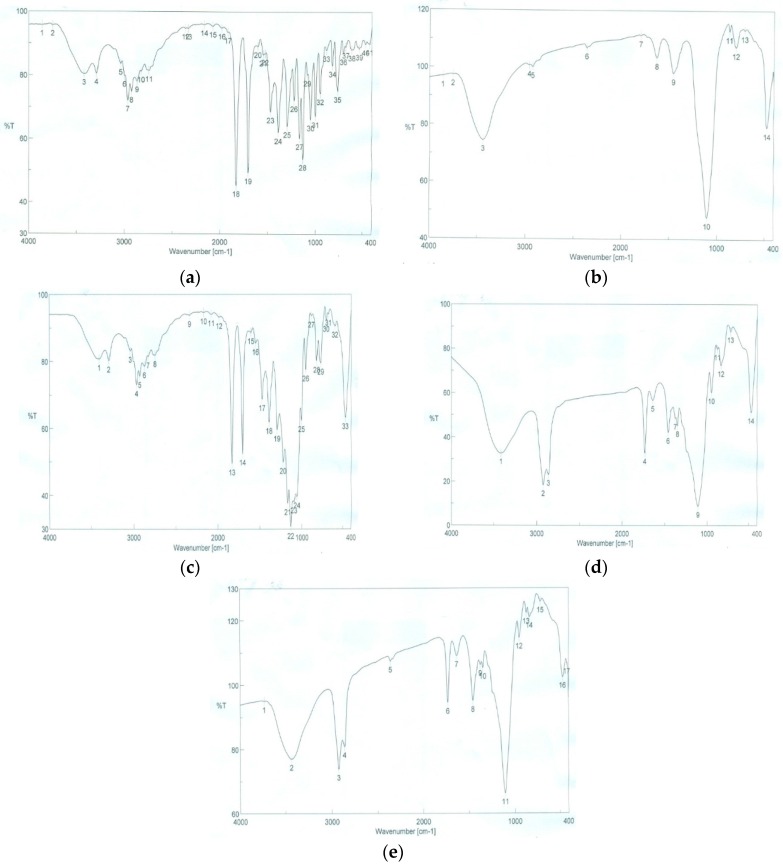
FTIR spectra of (**a**) Pure OLM; (**b**) Aerosil 200; (**c**) Physical mixture of OLM and Aerosil 200; (**d**) OLM S-SNEDDS formula (F3); (**e**) OLM S-SNEDDS formula (F4).

FTIR spectra are mainly used to determine interaction between the drug and any of the excipients used. The presence of interaction is detected by the disappearance of the important functional group of the drug. The IR spectrum of OLM revealed a characteristic peak at 3419 cm^−1^ for O–H group, a peak at 3290 cm^−1^ for N–H group and two characteristic sharp peaks at 1831 and 1706 cm^−1^ for two C=O groups. The spectrum of OLM also showed aromatic C=C stretching at (1550, 1532, 1474 cm^−1^) and *sp*^2^ C–H stretching at (3040, 3000 cm^−1^). In addition, the spectrum showed six peaks for C–O stretching at (1299, 1227, 1170, 1135, 1087, 1052 cm^−1^) as presented below). Similar peaks were observed in the spectra of physical mixture and prepared S-SNEDDS formulae (F3 and F4), along with absence of interfering peaks indicating there is no unwanted interaction between OLM and other used excipients in the study as shown in Figure 9.

#### 3.4.6. Drug Loading Efficiency

The amount of OLM present in the two optimized S-SNEDDS formulae was found to be within the USP limit. The drug loading efficiency was found to be 95.75 ± 0.65 for formula F3 and 92.30 ± 1.91 for formula F4. The drug content in S-SNEDDS was almost identical with the results obtained in liquid SNEDDS, so there was no change of percentage drug content after conversion of liquid to solid SNEDDS using spray drying technique. These results were in alignment with the high drug content found by Bhagwat et al. who prepared solid self-microemulsifying drug delivery system of telmisartan [36].

#### 3.4.7. In Vitro Drug Release Studies

**Figure 10:**
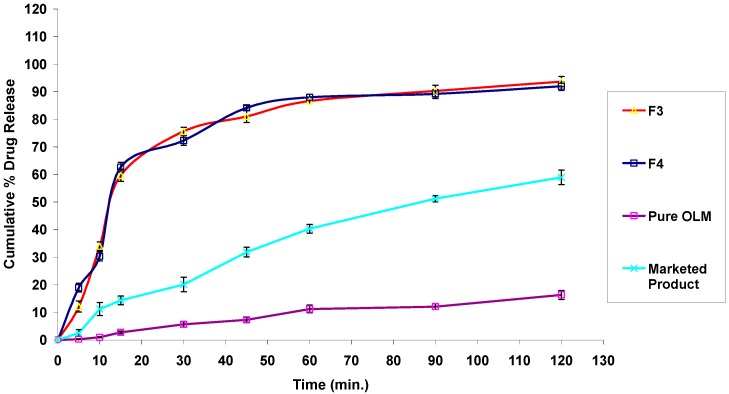
In vitro release profiles of OLM S-SNEDDS formulae (F3 and F4) compared with pure OLM and marketed product.

**Figure 11:**
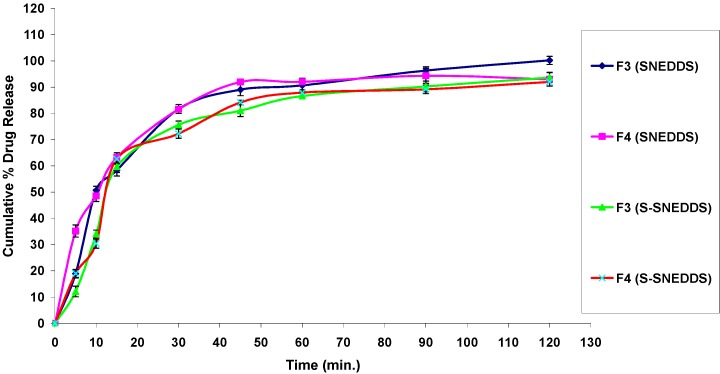
Comparison study of in vitro release profiles of OLM SNEDDS formulae and S-SNEDDS formulae (F3 & F4).

The percentage drug release from S-SNEDDS was found to be higher than that of pure OLM and marketed product as shown in Figure 10. Within the initial one hour of the in vitro release study, only 11.23% ± 1.42% and 40.33% ± 1.56% of OLM was dissolved from pure drug and marketed tablets, respectively, whereas the S-SNEDDS formulae showed improved release within the same time period. OLM dissolved and released from S-SNEDDS reached 86.64% ± 1.06% for formula F3, 87.96% ± 1.15% for formula F4 within one hour. The drug release study also indicates that the self-nanoemulsifying property of the formulation remains unaffected by the conversion of the liquid SNEDDS to the solid form as illustrated in Figure 11. It was also noticed that the release of OLM from S-SNEDDS was slightly lower than liquid SNEDDS. This might be attributed to the presence of adsorbent material which may delay the dissolution rate for a small extent. These findings were also compatible with Bhagwat et al. who found that the drug release profiles of the liquid SNEDDS showed no significant differences when compared to those of the solid SNEDDS, suggesting that the SNEDDS preserves a similar performance in emulsification regardless of the form (i.e., liquid or solid) [36].

#### 3.4.8. Pharmacokinetic Study

**Figure 12:**
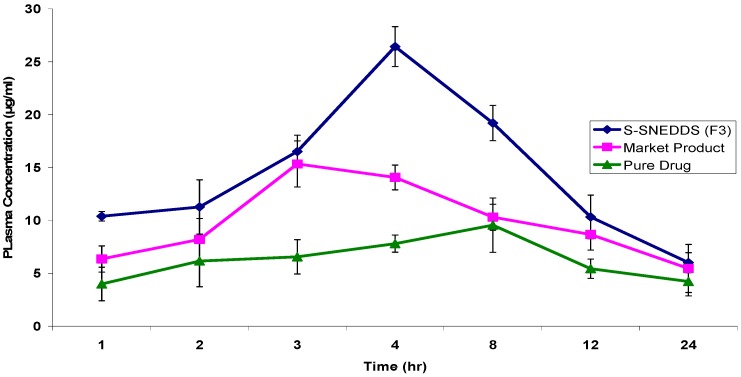
Plasma concentration-time profiles of OLM after oral administration of optimized S-SNEDDS formula (F3), Marketed product and pure drug in rats (*n* = 6).

To clarify the possible improvement in pharmacokinetic behavior of OLM, the plasma concentration-time curve profiles of OLM after the oral administration of optimized S-SNEDDS formula (F3) were compared to marketed products and drug in suspension as depicted in Figure 12. Formula F3 was selected for pharmacokinetic study due to its smallest particle size, great drug release profile as well as its higher drug loading efficiency.

As depicted in Table 11, the maximum concentration (*C*_max_) of OLM S-SNEDDS formula (F3) was 26.4320 ± 1.89 µg/mL, compared with marketed product which was 15.3365 ± 2.18 µg/mL and pure drug which was 9.5562 ± 2.37 µg/mL. The *C*_max_ was enhanced 1.72 and 2.77 fold as compared with marketed product and pure drug respectively. It was also observed that OLM S-SNEDDS formula (F3) showed high area under the curve value (299.7304 ± 4.15 μg·h/mL) in comparison with marketed product (208.3887 ± 6.47 μg·h/mL) and pure drug (143.2684 ± 3.59 μg·h/mL) indicating rapid absorption and higher bioavailability of OLM from S-SNEDDS formula (F3). The relative bioavailability of OLM in S-SNEDDS formula was about 1.44 fold compared with marketed product and about 2.09 fold compared to pure drug.

## 4. Conclusions

In this study, liquid SNEDDS was formulated and further developed into solid SNEDDS by a spray-drying technique using Aerosil 200 as the solid carrier. From this study, it was concluded that the prepared liquid SNEDDS was thermodynamically stable with good self-emulsification efficiency and having globule size in the nanometric range which may be physiologically stable. It was also concluded that S-SNEDDS preserved the self-emulsification performance of the liquid SNEDDS and gave a faster in vitro dissolution rate than the crude powder and marketed product. Furthermore, our results suggest that S-SNEDDS could be considered and further evaluated for the oral delivery of lipophilic poor soluble drugs for which an oral route of administration is desirable. In conclusion, self-emulsifying drug delivery systems represented a promising approach for the formulation of OLM. S-SNEDDS appeared to be an interesting approach to improving problems associated with oral delivery of OLM. Thus, S-SNEDDS can be considered as a new and commercially feasible alternative to current marketed OLM. Finally, the oral delivery of hydrophobic drugs can be made possible by S-SNEDDS, which have been shown to substantially improve the oral bioavailability.

## Figures and Tables

**Table 1:** Percent *w*/*w* Compositions of Optimized OLM Loaded SNEDDS Formulae.

Formula	OLM (mg)	Oil (% *w*/*w*)	Smix (% *w*/*w*)
F1	20	5	20
F2	20	5	40
F3	20	5	60
F4	20	5	80
F5	20	8.5	60
F6	20	8.5	80
F7	20	11.5	60
F8	20	11.5	80

**Table 2:** Emulsification Efficiency of Various Surfactants.

Surfactants	% Transmittance *	No. of Inversions
Cremophor RH40	99.47 ± 0.12	4
Cremophor S9	14.90 ± 0.66	17
Tween 20	98.17 ± 0.40	5
Tween 40	80.97 ± 1.01	11
Tween 60	74.93 ± 0.35	9
Tween 80	97.60 ± 0.26	15
Span 20	52.67 ± 0.75	17
Span 80	56.57 ± 0.50	13
Labrasol	44.87 ± 0.95	7

* Values are expressed as mean ± S.D., *n* = 3.

**Table 3:** Emulsification Efficiency of Various Cosurfactants.

Cosurfactants	% Transmittance *	No. of Inversions
PEG 200	99.33 ± 0.38	5
PEG 400	99.53 ± 0.12	4
PEG 600	94.43 ± 0.15	4
Transcutol HP	99.83 ± 0.06	3
Propylene glycol	98.80 ± 0.26	7
Glycerol	99.20 ± 0.10	15

* Values are expressed as mean ± S.D, *n* = 3.

**Table 4:** Thermodynamic Stability Studies of Various IRB Loaded SNEDDS Formulae.

Formula	Heat-Cool Cycles	Centrifugation Test	Freeze Thaw Cycles
F1	√	√	√
F2	√	√	√
F3	√	√	√
F4	√	√	√
F5	√	√	√
F6	√	√	√
F7	√	√	√
F8	√	√	√

Where (**√**) indicates the formula passed the test.

**Table 5:** Robustness to Dilution Results of Various OLM Loaded SNEDDS Formulae.

Formula	Distilled Water			0.1 N HCL			Phosphate Buffer pH 6.8		
	10	100	1000	10	100	1000	10	100	1000
F1	√	√	√	√	√	√	√	√	√
F2	√	√	√	√	√	√	√	√	√
F3	√	√	√	√	√	√	√	√	√
F4	√	√	√	√	√	√	√	√	√
F5	√	√	√	√	√	√	√	√	√
F6	√	√	√	√	√	√	√	√	√
F7	√	√	√	√	√	√	√	√	√
F8	√	√	√	√	√	√	√	√	√

Where (**√**) means stable formula which showed no phase separation or precipitation.

**Table 6:** Visual Observations of the Dispersibility Test for Various OLM SNEDDS Formulae.

Formula	Observations	Grade
F1	Rapidly forming clear emulsion	A
F2	Rapidly forming clear emulsion	A
F3	Rapidly forming clear emulsion	A
F4	Rapidly forming clear emulsion	A
F5	Rapidly forming clear emulsion	A
F6	Rapidly forming clear emulsion	A
F7	Rapidly forming clear emulsion	A
F8	Rapidly forming clear emulsion	A

**Table 7:** Self-Emulsification Time, % Transmittance, Particle Size, PDI, Zeta Potential and Drug Loading Efficiency of OLM SNEDDS Formulae.

Formula	Self-Emulsification Time * (s)	% T *	Particle Size (nm) *	PDI *	Zeta Potential (mV) *	Drug Loading Efficiency * (%)
F1	22.38 ± 0.77	99.14 ± 0.11	17.57 ± 0.26	0.076 ± 0.01	−4.23 ± 0.18	92.37 ± 0.75
F2	20.86 ± 1.26	99.30 ± 0.05	15.90 ± 0.32	0.057 ± 0.04	−3.62 ± 0.11	93.30 ± 0.86
F3	14.10 ± 0.75	98.15 ± 0.12	15.33 ± 0.19	0.044 ± 0.06	−2.63 ± 0.47	96.85 ± 1.23
F4	15.55 ± 0.85	98.40 ± 0.27	14.91 ± 0.12	0.056 ± 0.02	−4.10 ± 0.21	95.22 ± 2.22
F5	18.50 ± 1.38	97.97 ± 0.32	19.73 ± 0.15	0.058 ± 0.02	−2.88 ± 0.09	94.68 ± 1.91
F6	17.29 ± 1.31	99.60 ± 0.14	16.49 ± 0.21	0.036 ± 0.05	−3.34 ± 0.16	98.52 ± 1.45
F7	20.20 ± 1.88	99.18 ± 0.10	22.97 ± 0.44	0.077 ± 0.12	−5.13 ± 0.61	96.32 ± 1.88
F8	19.28 ± 1.13	98.33 ± 0.15	20.50 ± 0.39	0.241 ± 0.09	−4.47 ± 0.29	99.09 ± 0.56

* Values are expressed as mean ± S.D., *n* = 3.

**Table 8:** The Calculated Correlation Coefficients for the In Vitro Release of OLM from SNEDDS Formulae Employing Different Kinetic Orders or Systems.

Formula	Correlation Coefficients (*r*)				
	Zero Order	First Order	Second Order	Higuchi’s Diffusion Model	Hixson-Crowell Model
F1	0.8341	0.9053	0.9139	0.9574	0.8829
F2	0.7962	0.8869	0.8931	0.9486	0.8588
F3	0.8140	0.9126	0.7213	0.9709	0.9027
F4	0.7897	0.8632	0.8887	0.9823	0.8424
F5	0.8181	0.9036	0.7309	0.9758	0.9743
F6	0.9017	0.9655	0.9862	0.9874	0.9660
F7	0.8819	0.9501	0.7367	0.9804	0.9640
F8	0.9016	0.9701	0.9651	0.9963	0.9512

**Table 9:** Rank Order of OLM SNEDDS Formulae According to In Vitro Drug Release Data, Drug Loading Efficiency and Particle Size Analysis.

Formula	In Vitro Drug Release (1 h)	Drug Loading Efficiency	Particle Size	Total Rank Order	Conclusive Rank Order
F1	8	8	5	21	8
F2	5	7	3	15	6
F3	4	3	2	9	1
F4	3	5	1	9	1
F5	2	6	6	14	5
F6	6	2	4	12	3
F7	1	4	8	13	4
F8	7	1	7	15	6

**Table 10:** Micromeritic Properties of OLM Loaded S-SNEDDS.

Formula	F3	F4
Angle of Repose	27.64° ± 1.03°	22.79° ± 2.22°
Bulk Density (g/mL)	0.49 ± 0.02	0.51 ± 0.03
Tapped Density (g/mL)	0.59 ± 0.02	0.54 ± 0.01
Carr’s index (%)	14.32 ± 1.72	12.41 ± 0.95
Hausner’s ratio	1.22 ± 0.10	1.08 ± 0.08

**Table 11:** Pharmacokinetic Parameters of OLM After Oral Administration of Optimized S-SNEDDS Formula (F3), Marketed Product and Pure Drug in rats.

PK Parameters	S-SNEDDS (F3)	Marketed Product	Drug Suspension
*C*_max_ (µg/mL)	26.4320 ± 1.89	15.3365 ± 2.18	9.5562 ± 2.37
*t*_max_ (h)	4	3	8
AUC_0–24h_ (µg·h/mL)	299.7304 ± 4.15	208.3887 ± 6.47	143.2684 ± 3.59
*t*_1/2_ (h)	7.2489 ± 1.87	6.2488 ± 0.94	6.0896 ± 1.02
*K*_e_ (h^−1^)	0.0956 ± 0.002	0.1109 ± 0.004	0.1138 ± 0.001
